# Assessment of Dietary Iodine Intake in School Age Children: The Cross-Sectional ANIVA Study

**DOI:** 10.3390/nu10121884

**Published:** 2018-12-03

**Authors:** María Morales-Suárez-Varela, Isabel Peraita-Costa, Agustín Llopis-Morales, Agustín Llopis-Gonzalez

**Affiliations:** 1 Department of Preventive Medicine and Public Health, Food Sciences, Toxicology and Legal Medicine, School of Pharmacy, University of Valencia, Vicent Andres Estelles Avenue, Burjassot, 46100 Valencia, Spain; ivperaitacosta@hotmail.es (I.P.-C.); agustinllopis@gmail.com (A.L.-M.); agustin.llopis@uv.es (A.L.-G.); 2 Biomedical Research Consortium in Epidemiology and Public Health Network (CIBERESP), Monforte de Lemos Avenue, 3-5, Pavillion 11 Floor 0, 28029 Madrid, Spain

**Keywords:** iodine, children, deficiency, iodine sources, intake, nutrition

## Abstract

Iodine deficiency is one of the most important health problems in the world. It intervenes in the synthesis of thyroid hormones, which carry out important functions, so that a deficit of this mineral causes alterations of different kinds such as those related to growth. The objective of the present study was to know the prevalence of iodine deficit in the diet of Valencian children from 6 to 8 years old and their relationship with anthropometry. The analysis of the dietary intake was carried out through questionnaires. Thirteen schools participated in the study. The sample studied consists of 661 school children belonging to the Valencian Community, between 6 and 8 years of age: 298 boys and 363 girls. 79.12% of the children did not meet recommended daily iodine intakes. When comparing the groups of girls and boys with an inadequate intake, in general, girls show worse nutritional adequacy. When comparing the groups of girls and boys with sufficient iodine intake, no statistically significant differences were observed. No immediate effects of iodine deficiency on children’s anthropometry were observed. Intake of dairy products, fish and iodized salt is recommended, since they can contribute to the diet the iodine required to avoid a deficiency.

## 1. Introduction

Iodine is an essential element that is carried to the body through dietary intake. As an essential nutrient, iodine plays an important role in the synthesis of thyroid hormone (TH) [1]. The body of a healthy adult individual contains between 15 and 20 mg of iodine of which 70–80% is found in the thyroid [2,3].

Inadequate iodine intake affects the physical and mental development of millions of people around the world [4,5]. Until recently, the problem of iodine deficiency (ID) was basically focused on endemic goiter [6], but recent research has shown that there are other disorders, iodine deficiency disorders (IDD), that may lead to the increase in neonatal mortality and the number of miscarriages, congenital anomalies with permanent neuromotor damage, hearing defects and diminished intellectual capacity and growth [2,7]. In fact, the World Health Organization (WHO) considers ID the main global preventable cause of brain damage in the fetus and infant, and of psychomotor retardation in young children [8].

Even mild early ID may cause lasting and irreparable effects such as neonatal hypothyroidism, low intelligence, skeletal disorders including short stature, and other growth retardation issues [9,10]. Iodine is essential for the synthesis of TH [10] and TH aid in the regulation of fat metabolism, glucose utilization and protein production [11,12]. TH may also affect bone and muscle development in growing and developing children through the TH receptors present in bone cells and through the hypothalamic-pituitary-thyroid axis [13,14].

The latest data indicate that a total of 25 countries are those that are in situation of ID, so it remains a public health problem in the world. The WHO estimates that 740 million people are currently affected by goiter. Spain was considered by the WHO until 2004 as a country with ID. According to the latest data collected by the IGN, the average levels of iodinuria of the Spanish population are 117 μg/L, which indicates that it is at a level of sufficiency [15].

Childhood is a stage in which habits and eating patterns are acquired. A correct diet is necessary so that the growth is optimal and to avoid malnutrition or deficiency diseases, but nowadays, the prevention of future nutritional-based diseases and the establishment of healthy habits is also established as an objective. The assessment of nutritional status allows us to know if the nutrient supply is adequate according to age and sex [16].

In Spain, there is a multidisciplinary working group of the Spanish Society of Endocrinology and Nutrition (SEEN) on disorders related to ID, active for 25 years. Despite this, the application of public health prevention programs has been scarce. In 2005, the Ministry of Health and Consumer Affairs in collaboration with SEEN and United Nations International Children’s Emergency Fund (UNICEF) Spain, developed a campaign to encourage the replacement of common salt by iodized salt among pregnant women and the Health Ministries of Andalusia, Catalonia, Galicia, and Basque Country introduced the recommendation of the prescription of potassium iodide during pregnancy and lactation [17].

The objective of the present study is to determine the prevalence of compliance within school children from 6 to 8 years of age in the Valencian Community with the corresponding established Recommended Daily Intake (RDI) values of dietary iodine intake. 

This study was conducted according to the guidelines laid down in the Declaration of Helsinki and all procedures involving human subjects/patients were approved by the ethics committee of the Universitat de Valencia. Written informed consent was obtained from all subjects.

## 2. Methods

### 2.1. Population and Sample

The sample studied consisted of 661 schoolchildren belonging to the Valencian Community, between 6 and 8 years of age part of the ANIVA (Antropometria y Nutricion Infantil de Valencia) study, of which 298 were boys (45.08%) and 363 girls (54.92%).

Data collection began with the presentation to the schools of a formal letter and the authorization of the project by the Ministry of Education, Culture and Sport and the University of Valencia. Subsequently, a meeting was arranged with the director and parents’ association to present the study. After approval, the school was considered a participating center.

**Figure 1:**
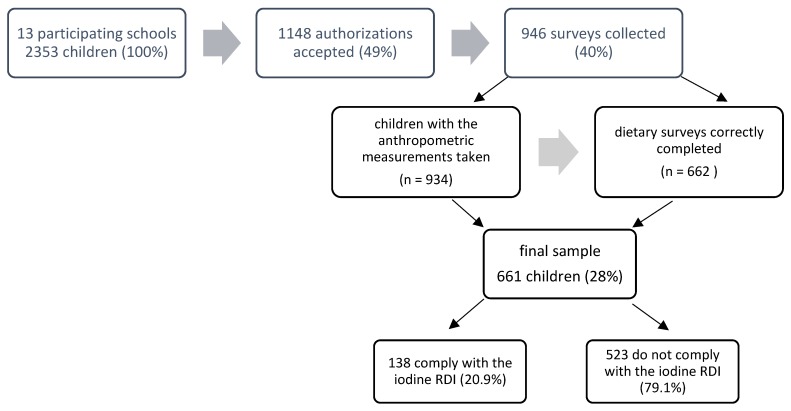
Flow diagram of the study population. RDI: Recommended Daily Intake.

A total of 13 schools participated in the study. The schools were randomly selected within the Valencian Community and all schools contacted agreed to participate. A letter was sent to the parents or guardians with the informed consent attached. After consent was returned, the survey to be completed by parents or guardians was given. This survey consisted of a description of the study, a brief questionnaire aimed at knowing the level of studies of the parents or guardians and the physical activity of the student, as well as a record of the dietary intake (Figure 1). 

### 2.2. Anthropometric Measures

The anthropometric data collected were the weight, using a Soehnle electronic scale model 63,760 with a capacity of 150 kg and a precision of 0.1 kg, the height, through a Seca 213 portable stadiometer that allows measuring up to 250 cm with division in millimeters and waist-hip circumference through a tape measure.

Once the values obtained for each individual were recorded, the WHO Anthro software version 3.2.2 of January 2011 (World Health Department of Nutrition, Geneva, Switzerland), the Body Mass Index (BMI) for the age (z-score), the weight for age (z-score) and height for age (z-score). In this way we obtain a comparison between the real values obtained in the measurement of the child and those values of growth that would be optimal. The standard deviation values used in the study are governed by the WHO International Child Growth Standards [18].

### 2.3. Diet Assessment

The analysis of the dietary intake was carried out through questionnaires of food intake records consumed, previously validated for a pediatric population, over the course of three days, one of which was a holiday. The analysis of the data recorded in the questionnaires was made through the DIAL software for assessing diets and food calculations (Department of Nutrition (UCM) & Alce Ingeniería, S.L. Madrid, Madrid, Spain.), which provides a detailed assessment of the diet by generating macro and micronutrient data corresponding to each individual, these would be included in a database for analysis statistical later. The software has been previously validated and the nutrient database was last updated in August 2018. The database has a table of nutritional composition of more than 800 foods, which contains a vast information on the composition of energy, proteins, lipids, carbohydrates, fiber, minerals, vitamins, cholesterol, fatty acids, amino acids, etc. (up to a total of about 140 different components) of the most common foods. The information of a product can be located not only by the most common name, but also incorporates an extensive list of local, regional or national names, up to more than 2000 different names, to which must be added the possibility of searching through the scientific name and also its equivalence in English. It also uses a table of homemade measurements, with about 1860 entries, with units and rations usually used. Therefore, nutrient intake is estimated using both the type of food consumed and the quantity. The software also allows to estimate the recommended intakes of energy and nutrients according to the individual characteristics of a person: age, sex, weight, physical activity. The questionnaire used was specifically designed to be used with the DIAL software by the Universidad Complutense Department of Nutrition who are one of the developers of the software.

Iodine intake has been assessed using a dietary intake questionnaire in order to be able to compare these values with the established recommended dietary intake values. This study is part of a larger study (ANIVA) centered on the dietary pattern and habits of the sample population whose results are compiled and derive into nutritional intervention when required.

The tables for the Spanish population of 6 to 8 years old prepared by the Federación Española de Sociedades de Nutrición, Alimentación y Dietética (FESNAD) in 2010 were used for RDI comparison [19]. RDI is the average daily dietary intake level that is sufficient to meet the nutrient requirements of nearly all (97–98 percent) healthy individuals in a particular life stage and gender group while Estimated Average Requirement (EAR) is the daily nutrient level estimated to meet the requirements of half the healthy individuals in a particular life stage and gender group [19]. When an RDI cannot be determined Adequate Intake (AI) is used and defined as the average daily nutrient intake level based on observed or experimentally-determined approximations or estimates of nutrient intake by a group (or groups) of apparently healthy people that are assumed to be adequate [19].

### 2.4. Physical Activity

The questionnaire used was based on the National Health Survey 2011–2012 conducted by the Ministry of Health, Social Services and Equality of Spain (2013).

The frequency of physical activity carried out by the child was assessed according to: does not perform physical activity; performs physical exercise less than once a month; performs physical exercise one or more times a month but less than once a week; performs physical exercise weekly for a time of less than 2 h; or performs physical exercise weekly for 2 h or more.

### 2.5. Socio-Educational Level

The child’s environment was assessed through questions about the education of the parent/guardian/tutor which was classified into: without studies; primary studies, secondary studies; or university studies and postgraduate university studies.

### 2.6. Statistical Analysis

The results obtained were analyzed and are reflected in the present study, expressing the values of the means together with their standard deviations for quantitative variables and as a percentage for qualitative ones.

Pearson’s ANOVA and Chi-square Test were applied to determine the possible significant differences of the study groups, the first one being applied to the quantitative variables and the second one to the qualitative ones. The computer programs used for the statistical analysis were SPSS (IBM Corp., Armonk, New York, United States of America) and Epidat (Servicio de Epidemiología de la Dirección Xeral de Saúde Pública de la Xunta de Galicia, Santiago de Compostela, A Coruña, Spain). 95% (*p* ≤ 0.05) was taken as a level of statistical significance.

## 3. Results

The basic characteristics stratified by iodine RDI compliance and sex are summarized in Table 1. A total of 79.1% of the sample had inadequate iodine intake, 56.2% of girls and 43.8% of boys. Schoolchildren who do not comply with the iodine RDI are those who perform an inappropriate physical activity practice (*p* = 0.006). In addition, girls who do not meet the iodine RDI perform less physical activity than boys (*p* = 0.001). However, no statistically significant differences were found according to gender, BMI or educational level of the father/mother.

Table 2 summarizes the nutritional inadequacy according to iodine intake and sex. Average iodine intake in children with adequate intake was 174.9 ± 168.2 μg/day, in contrast, that of children with inadequate intake was 87.9 ± 17.7 μg/day. When comparing groups of girls and boys with inadequate iodine intake, in general, girls show a worse nutritional adequacy. On the other hand, when comparing the groups of girls and boys with sufficient iodine intake, no statistically significant differences were observed between the sexes. 

Table 3 analyzes the data regarding the percentage of intake of foods studied by gender and with respect to the compliance or not of the RDI of iodine. In the results it is observed, in spite of not finding statistically significant differences, within the children who comply with the RDI of the iodine, boys are the ones who fulfill a regular intake of fish and dairy products, while, in the schoolchildren who do not comply with RDIs of iodine girls are who meet the regular intake of fish and dairy.

## 4. Discussion

The US Institute of Medicine recommends the intake of 90 μg daily of iodine for children under 4–8 years of age [20] and WHO 120 μg daily of iodine for those aged 6–12 years [5]. On the other hand, the EFSA estimates that adequate iodine intake for children between 4 and 10 years of age is 90 μg/day [21]. The results obtained in the study warn of an insufficiency of the iodine intake of 79.1% of the Valencian children between 6 and 8 years studied when the 90 μg/day cutoff is used given that it is the recommended intake according to age, sex, weight, and physical activity of the population studied as determined by FESNAD.

Daily ioduria excretion is considered the most reliable measurement of iodine nutritional status of an individual [22,23,24], however, previous studies, using different dietary iodine intake assessment methods than the present study, have shown a correlation between ioduria levels and dietary intake assessment results [25,26]. The reason iodine nutritional status is not measured using ioduria levels here is that the present work is part of a larger observational research study centered on the nutritional intake of a pediatric population where dietary pattern and habits are the main focus since one of the main objectives is to be able to establish adequate dietary intervention recommendations. 

Due to the lack of similar studies assessing dietary iodine intake, the results of this study are compared to those of studies in which ioduria was assessed. Ioduria studies are carried out in schoolchildren because they constitute a group congregated in the same place and are representative of the general state of ID. experts at an international level have determined that they are the tracer group to measure the impact of interventions for the eradication of IDD [27]. The fluctuations in concentrations within a population reflect changes in iodine nutritional status which may result from changes in societal and commercial practices while fluctuations within an individual can be the result of differences in daily iodine intake. water consumption and/or physical activity [22].

Studies have been conducted in different areas of Spain. which reflect iodine sufficiency in the majority of the school population. A study carried out in Lleida in 6-year-old children shows a median ioduria of 234.41 μg/L [28]. In a study in the Basque Country, the median iodine ranged between 131-161 μg/L for the same age range [29]. The Tirokid study, which includes 11 Spanish communities, determined in 2016 an adequate level of iodine in 44.1% of children aged 6 to 7 years and a median iodine level of 173 μg/L. Within the Valencian Community, one study showed a median ioduria of 155 μg/L in children aged 6–14 years and ranged from 155–165 μg/L in the age range of 6–8 years [30]. Another one carried out a year later in children from 6 to 11 years old indicated that the median level of iodide was 188 μg/L, and among school children from 6 to 8 years old the values of this were between 137.5–204 μg/L [31]. The children of our sample (20.88%) were below the adequacy iodide levels of schoolchildren in the studies carried out in Alicante (86.5%) [31], Lleida (67.49%) [28], or the Tirokid study (44.1%) [30].

It was considered important to analyze the students’ environment in order to determine possible external factors could affect nutritional intake. The educational level of the parents was predominantly secondary studies, however, among the group of children who meet the iodine RDI there were more parents with university education. The mothers had more schooling in relation to the fathers, especially those who met the iodine RDI. In a previous study, a parental low level of education was associated with insufficient iodine intake while a high level of education was associated with higher intake of both iodized salt and milk [32]. The “Tirobus project”, carried out on the general population, did not show significant differences between iodine intake according to education and occupation [33].

Physical activity is another factor to consider. In the present study, schoolchildren who did not comply with the iodine RDI were those who performed an inappropriate level of physical activity. In addition, girls who do not meet the iodine RDI performed less physical activity than boys. In children over 5 years of age, daily physical activity of at least one hour and of moderate to vigorous intensity is recommended. The promotion of outdoor physical activity and limiting the use of the screen is also intended [34].

In our study, among school children that met the iodine RDI and those who did not, there were significant differences in energy intake and the majority of macro and micronutrients studied. Intakes were unbalanced and the energy intake exceeded the recommendations for this age group. If this continues, it could result in an inadequate supply of micronutrients of great relevance in metabolism, development, and growth, and, in turn, an increase in the possibility of obesity and chronic cardiovascular diseases.

Among the foods that contribute the most iodine are seafood and dairy products. Fish intake in most of our study sample was less than 2 times a week, similar to the results obtained in a 2015 study [31]. In the Tirokid study, median fish intake was twice a week [32]. In a 2010, a relationship was established between fish intake and attendance at the school canteen, with a lower intake of fish among children who do not go to the canteen. They associate this relationship with the fact that the school canteen ensures the contribution of fish since the menu is made following nutritional recommendations [28]. Regarding dairy products, more than 90% of the schoolchildren did not consume them at breakfast. The children in the group who complied with the iodine RDI were those who consumed more of these products at breakfast. The intake of dairy products in meals other than breakfast seemed to occur more frequently, however, most students would have difficulty ingesting the 2–3 currently recommended dairy rations that would comprise their daily iodine intake [35]. The milk intake values of our sample differ from those of the Tirokid study, since 81% of the children in that study ingested 2 or more dairy products a day, and 80% ate cheese at least once a week [32]. As for Lleida schoolchildren, 90.65% drank milk daily, and it was determined that the risk of iodine deficit among those who only consumed milk one day a week was greater than those who did it daily [28]. Among the students in our study, we did not find significant differences between the groups regarding the intake of fish, cheese, and yoghurts or dairy products at breakfast, so possibly the adequacy or not of the iodine RDI in our sample was given to the intake of milk in other foods not reported, or the intake of foods that provide iodine. It is also possible to consider the different cooking methods since the process of food production reduces its iodine content in different degrees, being on average 20% in the case of frying, 23% in roasting, and 58% in boiling [36].

As we have mentioned before, the composition in iodine of different foods is affected by different elements, therefore knowing the composition in this element in fortified foods or those that benefit from the contribution of animal iodine that produces them can facilitate the adequacy of the population to the established recommendations.

In the study carried out in 2015, an average concentration of iodide in UHT milk in Spain was determined which fluctuated throughout the year. They calculated that a ration of milk provided around 50 μg/day of iodine, 42% of what children from 6 to 8 years would need. Although no statistically significant differences were found, the skimmed milk possessed the most iodine, then the semi-skimmed, and finally the whole milk [37].

On the other hand, iodized salt in Spain is a fortified product chosen for its daily intake as a vehicle for the supply of iodine. The intake of half a tablespoon of coffee or of iodized salt a day provides 150 μg of iodine [38]. Therefore, the WHO recommends that in order to achieve an adaptation to iodine intake 90% of households should consume iodized salt, as well as the universalization of iodized salt.

## 5. Limitations

One of the main limitations of the study is that while the difference in mean iodine intake between the RDI compliant and non-compliant group is large—87.0 µg for boys and 65.2 µg for girls—the non-compliant group almost meets the RDI which for iodine in this age group is 90 μg/day. The mean intakes for non-compliant boys and girls are 97.6% and 93.9% of the RDI respectively. The fact that the non-compliant group is so close to meeting the RDI could account in part for the lack of significant differences in the anthropometric values of the children studied.

Another limitation is derived from the selected values of iodine in the foods which may impact the estimated iodine intake. These values were not selected by the authors and are a proprietary part of the DIAL software package which could not be evaluated by the authors. However, the software is validated for dietary intake estimation, has been updated very recently and therefore its reliability is not in question. 

Finally, the results in this study need to be interpreted with caution given that no biochemical biomarkers of iodine status were collected and the estimated intake values could not be contrasted. Future studies should aim to include both dietary and biochemical data to present a more complete picture of iodine status. 

## 6. Conclusions

From these results it has been concluded that 79.1% of the Valencian children aged 6 to 8 years studied do not comply with the iodine RDI. The intake of foods with a high iodine content is below the recommendations, therefore, the institutions should make families aware of the need for the intake of iodized salt for the prevention of pathologies derived from ID. In Spain, with an adequate intake of dairy products and iodized salt, the levels recommended for children of school age could be achieved. The prevention of ID should be done especially at growing ages, given the importance of this mineral in the formation of body structures.

## Figures and Tables

**Table 1:** Anthropometric characteristics of schoolchildren in relation to iodine intake.

Variables	RDI Compliant (*n* = 138; 20.9%)					RDI Non-Compliant (*n* = 523; 79.1%)					*p*-Value
	Boys (*n* = 69; 50%)		Girls (*n* = 69; 50%)		*p*-Value (Boys vs. Girls)	Boys (*n* = 229; 43.8%)		Girls (*n* = 294; 56.2%)		*p*-Value (Boys vs. Girls)	
	Mean	SD	Mean	SD		Mean	SD	Mean	SD		
Age (years)	7.5	1.1	7.5	1.1	0.917	7.5	1.1	7.6	1.1	0.350	0.642
Weight (kg)	29.9	7.2	29.8	7.2	0.928	30.7	8.2	31.2	8.5	0.508	0.126
Height (m)	1.3	0.1	1.3	0.1	1	1.3	0.1	1.3	0.1	1	0.493
Weight z-score	0.9	1.1	0.7	1.0	0.345	1.0	1.2	1.0	1.2	0.634	0.141
Height z-score	0.7	1.0	0.5	1.1	0.321	0.7	1.0	0.7	1.1	0.831	0.353
BMI z-score	0.8	1.2	0.7	1.0	0.677	0.8	1.4	0.8	1.4	1	0.364
**Mothers’ educational level (%)**											
Low	17.5		13.0		0.477	21.8		26.5		0.215	0.061
Average	42.0		42.0		1	45.5		37.8		0.077	0.368
High	40.5		44.9		0.605	32.7		35.7		0.479	0.255
**Fathers’ educational level (%)**											
Low	37.7		36.2		0.860	41.5		38.1		0.431	0.789
Average	40.6		43.5		0.730	38.9		38.4		0.920	0.882
High	21.7		20.3		0.834	19.7		23.5		0.293	0.755
**Level of physical activity (%)**											
Inadequate	36.2		47.8		0.167	40.2		56.1		0.001	0.006
Adequate	63.8		52.2		0.167	59.8		43.9		0.001	0.001

RDI. Recommended Daily Intake; Mean and SD were compared with the ANOVA Test and % with the chi-square test; *p*-Value < 0.05 is considered statistically significant. BMI: Body Mass Index.

**Table 2:** Nutritional insufficiency in relation to iodine and gender consumption.

Nutrients	RDIs	RDI Compliant (*n* = 138; 20.9%)					RDI Non-Compliant (*n* = 523; 79.1%)					*p*-Value (Total)
		Boys (*n* = 69; 50%)		Girls (*n* = 69; 50%)		*p*-Value (Boys vs. Girls)	Boys (*n* = 229; 73.8%)		Girls (*n* = 294; 56.2%)		*p*-Value (Boys vs. Girls)	
		Mean	SD	Mean	SD		Mean	SD	Mean	SD		
Iodine (μg)	<EAR	174.9	168.2	149.8	34.4	0.227	87.9	18.7	85.5	13.8	0.013	0.001
Iodine (μg)	<EAR	138.0 *	33.0 **	136.0 *	35.0 **	0.620	88.0*	25.0 **	85.3 *	30.0 **	0.069 ***	0.001
Total energy (kcal)	<EAR	2587.3	401.9	2533.6	504.0	0.760	2204.8	383.6	2071.6	363.2	0.001	0.001
Proteins (g)	>EAR	113.5	18.5	110.6	24.3	0.433	89.6	22.6	84.5	20.7	0.006	0.001
Carbohydrates (g)	<EAR	257.9	47.4	248.4	60.9	0.307	228.5	39.5	210.2	37.7	0.001	0.001
Fat (g)	>EAR	117.9	24.3	118.1	24.4	0.950	99.4	23.1	95.56	23.6	0.062	0.001
Cholesterol (mg)	>EAR	433.7	119.6	434.8	109.7	0.955	320.6	101.5	299.6	93.6	0.014	0.001
Dietary fiber (g)	<AI	20.2	5.7	19.0	5.4	0.190	18.2	10.9	16.7	5.3	0.039	0.003
Vitamin B1 (mg)	<EAR	1.7	0.5	1.7	0.8	0.857	1.5	0.5	1.4	0.5	0.101	0.001
Vitamin B2 (mg)	<EAR	2.3	0.6	2.5	2.4	0.410	1.9	0.5	1.7	0.5	0.001	0.001
Niacin (mg)	<EAR	46.0	9.8	43.9	10.8	0.244	34.7	9.2	32.9	8.3	0.013	0.001
Vitamin B6 (mg)	<EAR	2.5	0.7	2.7	2.4	0.396	2.1	0.6	2.0	0.6	0.044	0.001
Folic acid (μg)	<EAR	282.5	94.6	280.4	94.6	0.893	237.9	78.3	226.5	80.9	0.105	0.001
Vitamin B12 (μg)	<EAR	8.2	3.6	7.9	3.4	0.582	6.8	7.5	5.8	3.2	0.041	0.001
Vitamin C	<EAR	113.0	53.1	114.3	60.3	0.891	100.5	50.3	90.3	43.4	0.012	0.001
Biotin (μg)	<EAR	35.1	8.8	33.8	8.0	0.345	28.2	10.3	25.6	8.6	0.001	0.001
Ac. Pantothenic (mg)	<EAR	6.8	1.1	6.8	1.7	0.966	5.6	1.4	5.1	1.3	0.001	0.001
Vitamin A (μg)	<EAR	1174.4	599.0	1246.2	888.1	0.578	1113.5	1454.8	864.0	437.9	0.005	0.011
Vitamin D (μg)	<EAR	4.4	2.7	4.6	3.7	0.742	3.2	3.5	3.2	2.4	0.969	0.001
Vitamin E (mg)	<EAR	10.6	3.5	10.0	4.1	0.374	8.0	2.9	7.9	3.1	0.908	0.001
Calcium (mg)	<EAR	1166.4	234.2	1187.1	378.6	0.699	926.1	226.4	873.2	229.9	0.008	0.001
Iron (mg)	<EAR	15.8	5.2	15.1	4.6	0.405	13.3	4.8	12.3	3.9	0.010	0.001
Magnesium (mg)	<AI	343.4	77.5	338.9	79.9	0.736	276.2	74.4	263.5	60.2	0.031	0.001
Zinc (mg)	<EAR	11.3	1.8	11.0	2.5	0.471	9.3	2.4	8.8	2.1	0.004	0.001
Selenium (mg)	<EAR	154.5	39.3	143.6	40.6	0.111	107.3	30.1	103.9	28.9	0.080	0.001
Sodium (mg)	<EAR	2921.7	733.8	2945.4	1060.7	0.878	2209.0	737.7	2054.3	637.6	0.010	0.001
Potassium (mg)	<EAR	3356.8	544.4	3396.8	797.3	0.731	2725.5	621.2	2684.0	2088.8	0.771	0.001
Fluoride (μg)	<AI	376.3	297.9	344.0	195.6	0.453	335.1	317.8	321.6	289.2	0.610	0.243
Phosphorus (mg)	<EAR	1800.8	275.5	1808.5	390.9	0.894	1415.3	269.6	1327.4	285.1	0.001	0.001

AI: Adequate intake; TEV: Total energy value; EAR: Estimated average requirement; RDI: Recommended daily intake; EAR values: carbohydrates (50–60% TEV); proteins (10–15% TEV); calcium (20 mg/g); fat (30–35% TEV); thiamin (0.8 mg/day); riboflavin (1.2 mg/day); niacin (12 mg/day); vitamin B6 (1.4 mg/day); biotin (12 μg/day); vitamin B12 (1.5 μg/day); folic acid (200 μg / day); vitamin C (55 mg/day); vitamin A (400 μg/day); vitamin D (5 μg/day); vitamin E (8 mg/day); calcium (800 mg/day); phosphorus (700 mg/day); iron (9 mg / day); zinc (10 mg / day); iodine (90 μg/day); selenium (30 μg/day); AI: pantothenic acid (3 mg / day); magnesium (180 mg/day); fluorine (1000 μg/day); A consumption of carbohydrates <50% TEV. proteins> 15% TEV. and fats> 35% TEV was considered inadequate; Mean and SD were compared with the Student’s *t*-test; *p*-Value <0.05 is considered statistically significant. * Median. ** Interquartile range. *** Median and interquartile range were compared with the Mann–Whitney *U* test; *p*-Value < 0.05 is considered statistically significant.

**Table 3:** Consumption of fish and dairy products of the sample studied by gender and with respect to the suitability of the iodine RDI.

Consumption of Fish and Dairy Products		RDI Compliant (*n* = 138; 20.9%)			RDI non-Compliant (*n* = 523; 79.1%)			*p*-Value (Compliant Boys vs. Non-Compliant Boys)	*p*-Value (Compliant Girls vs. Non-Compliant Girls)
		Boys (*n* = 69; 50%)	Girls (*n* = 69; 50%)	*p*-Value (Compliant vs. Non-Compliant)	Boys (*n* =229; 73.8%)	Girls (*n* = 294; 56.2%)	*p*-Value (Compliant vs. Non-Compliant		
		%	%		%	%			
Regular consumption of fish (at least 2 or 3 times / week)	Compliant	20.3	15.9	0.659	19.2	21.1	0.661	0.843	0.337
	Non-compliant	79.7	84.1		80.8	78.9			
Consumption of dairy products at breakfast	Compliant	8.7	4.3	0.493	3.1	6.1	0.147	0.094	0.778
	Non-compliant	91.3	95.7		96.9	93.9			
Consumption 2 yogurts and / or 40 g of cheese per day	Compliant	30.4	29.0	1.000	19.7	23.8	0.288	0.058	0.370
	Non-compliant	69.6	71.0		80.3	76.2			

RDI: Recommended Daily Intake; % were compared with the chi-square test; *p*-Value <0.05 is considered statistically significant.
